# How Should Clinical Wound Care and Management Translate to Effective Engineering Standard Testing Requirements from Foam Dressings? Mapping the Existing Gaps and Needs

**DOI:** 10.1089/wound.2021.0173

**Published:** 2023-11-03

**Authors:** Amit Gefen, Paulo Alves, Dimitri Beeckman, Breda Cullen, José Luis Lázaro-Martínez, Hadar Lev-Tov, Bijan Najafi, Nick Santamaria, Andrew Sharpe, Terry Swanson, Kevin Woo

**Affiliations:** ^1^ Department of Biomedical Engineering, Faculty of Engineering, Tel Aviv University, Tel Aviv, Israel.; ^2^ Centre for Interdisciplinary Research in Health, Catholic University of Portugal, Porto, Portugal.; ^3^ Skin Integrity Research Group (SKINT), University Centre for Nursing and Midwifery, Ghent University and Swedish Centre for Skin and Wound Research, School of Health Sciences, Örebro University, Örebro, Sweden.; ^4^ RedC Consultancy, Bradford, United Kingdom.; ^5^ Diabetic Foot Unit, Universidad Complutense de Madrid, Madrid, Spain.; ^6^ Dr. Phillip Frost Department of Dermatology and Cutaneous Surgery, University of Miami Hospital Miller School of Medicine, Miami, Florida, USA.; ^7^ Interdisciplinary Consortium on Advanced Motion Performance (iCAMP), Michael E. DeBakey Department of Surgery, Baylor College of Medicine, Houston, Texas, USA.; ^8^ School of Health Sciences, University of Melbourne, Melbourne, Victoria, Australia.; ^9^ Podiatry Department, Salford Royal NHS Foundation Trust, Salford Care Organisation, Salford, United Kingdom.; ^10^ Nurse Practitioner, Warrnambool, Victoria, Australia.; ^11^ School of Nursing, Queen's University, Kingston, Ontario, Canada.

**Keywords:** treatment, laboratory testing methods and standards, test fluid, fluid handling and retention, exudate management

## Abstract

**Significance:**

Wounds of all types remain one of the most important, expensive, and common medical problems, for example, up to approximately two-thirds of the work time of community nurses is spent on wound management. Many wounds are treated by means of dressings. The materials used in a dressing, their microarchitecture, and how they are composed and constructed form the basis for the laboratory and clinical performances of any advanced dressing.

**Recent Advances:**

The established structure/function principle in material science is reviewed and analyzed in this article in the context of wound dressings. This principle states that the microstructure determines the physical, mechanical, and fluid transport and handling properties, all of which are critically important for, and relevant to the, adequate performances of wound dressings.

**Critical Issues:**

According to the above principle, once the clinical requirements for wound care and management are defined for a given wound type and etiology, it should be theoretically possible to translate clinically relevant characteristics of dressings into physical test designs resulting specific metrics of materials, mechanical, and fluid transport and handling properties, all of which should be determined to meet the clinical objectives and be measurable through standardized bench testing.

**Future Directions:**

This multidisciplinary review article, written by an International Wound Dressing Technology Expert Panel, discusses the translation of clinical wound care and management into effective, basic engineering standard testing requirements from wound dressings with respect to material types, microarchitecture, and properties, to achieve the desirable performance in supporting healing and improving the quality of life of patients.

**Figure:**
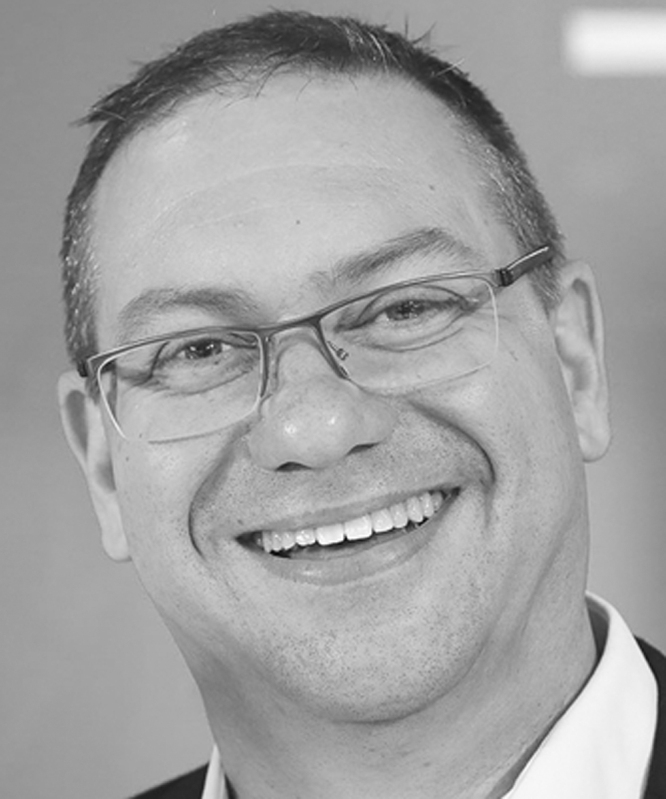
Amit Gefen, PhD

## BACKGROUND

### Scope and significance

Wounds remain one of the most important, expensive, and common medical problems, and many wounds are treated using dressings. The materials that make an advanced wound dressing, their microarchitecture, and how they are composed and constructed form the basis for its laboratory and clinical performances. This multidisciplinary review article discusses the translation of clinical wound care and management into effective, basic engineering standard testing requirements from wound dressings with regard to material types, microarchitecture, and properties, to achieve the desirable performance in supporting healing and improving the quality of life of patients.

### Translational relevance

The established structure/function principle in material science states that the microstructure determines the physical, mechanical, and fluid transport and handling properties, all of which are critically important for the adequate performances of advanced wound dressings. Accordingly, once the clinical requirements for wound care are defined for a given wound type and etiology, it should be theoretically possible to translate the clinically relevant characteristics of dressings into physical test designs resulting specific metrics of materials, mechanical, and fluid transport and handling properties, all of which should be determined to meet the clinical objectives and be measurable through standardized bench testing.

### Clinical relevance

Clinicians should adopt critical thinking concerning dressing technologies and specifications. Laboratory test data should be routinely requested from manufacturers to verify that the dressing being considered is capable of managing the exudate fluids that are relevant to the wound etiologies to be treated, for example, the expected exudate volumes, flow rates, and viscosities.

Requiring manufacturers to agree upon and implement standardized, clinically relevant test methods, resulting in peer-reviewed, published test data will facilitate informed selection of the safest and best performing dressings. Clinically relevant testing standards would also ultimately allow objective, standardized, and quantitative comparisons between dressing brands, thereby optimizing personalized treatment decisions.

### The critical role of laboratory test standards in dressing performance evaluations

Wounds and skin injuries of all types, including traumatic injuries and burns, surgical wounds and hard-to-heal (including chronic) wounds, such as pressure ulcers/injuries, venous leg ulcers, and diabetic foot ulcers, are one of the most important, impactful, expensive, and common medical problems.

For example, up to approximately two-thirds of community nursing time is spent on the provision of wound care and management, the majority of which is due to delayed healing.^1–3^ All serious wounds and some of the mild ones are treated by means of dressings. The materials currently used in modern wound dressings, their microarchitecture, and how they are composed and constructed in a dressing structure form the basis for the performance of dressings in contemporary, clinical wound care.

**Figure 1.**
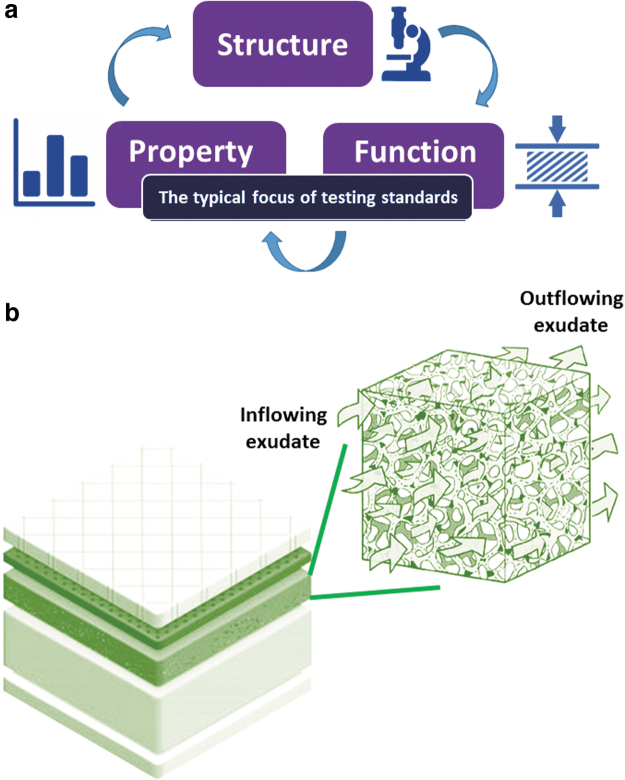
The established structure–function principle in material science is that the microstructure determines the properties, such as the mechanical and fluid handling characteristics **(a)**. For wound dressings, “function” encompasses the mechanical, fluid transport, and retention properties (altogether). The focus of the vast majority of the existing testing standards for wound dressings is on the properties and function of the tested dressing products, not their structure or microstructure. In a multilayer foam dressing, for example, each layer of the dressing has its own set of the above properties, and accordingly, the “function” of the whole dressing structure is determined by the contribution of each of the material components, for example, to the effective permeability of the dressing structure **(b)**.

**Figure 2.**
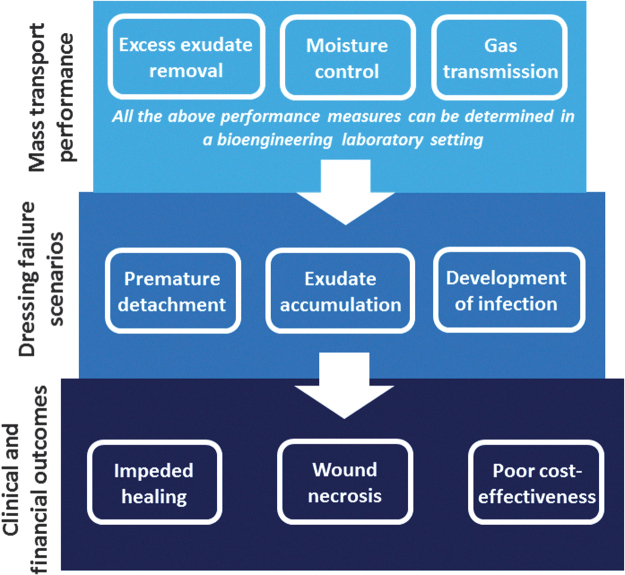
The fluid handling (or mass transport) performance of a certain wound dressing (as detailed in Table 1), which can be measured in a bioengineering laboratory setting, determines the likelihood of that dressing to successfully manage exudates, or alternatively, to fail in the more challenging clinical scenarios requiring, for example, the absorbency of high volume of exudation or of a viscous exudate; treatment of infected wounds; and protection of fragile periwound skin. Hence, the mass transport performance of the dressing eventually determines both the patient and the financial outcomes.

The established structure/function principle in material science states that the microstructure determines the physical, mechanical, and fluid transport and handling properties of a given dressing product (Fig. 1). All these properties are imperative and relevant to the laboratory testing of wound dressings and, more importantly, are critical to the clinical performance of dressings in supporting wound healing (Fig. 2).

**Table 1.** The primary clinical roles and functions of adequately performing wound dressings and the associated requirements from dressing materials and structures

Fundamental Clinical Requirements for Wound and Skin Protection and Repair			Other Requirements
Physical and Engineering		Biological	
Fluid Handling	Mechanical Behavior		
Effectively absorb a variety of wound exudates (*e.g.*, having different viscosities) while maintaining an optimally moist wound environment	Be compliant (*i.e.*, flexible) to conform to various contours of the body surface	Not be toxic, sensitizing, allergenic, or otherwise irritating to the wound area; not abnormally change the skin pH	Be acceptable in appearance to patients, family members, health care professionals, and others
Effectively retain absorbed exudates unloaded and under gravity, bodyweight, or any sustained or sudden external forces	Be reasonably strong and stiff to mechanically protect the wound but not rigid; to not abrade the wound	Be sterile	Have long storage life across a wide range of conditions
Effectively release retained fluids into the environment through the backing material/film as vapor, to facilitate additional absorbency	Not release any particulates or debris into the wound bed	Resist the penetration of bacteria, viruses, and fungi through the dressing into the wound, or their escape from the wound	Have low inflammability to keep patients safe near fire sources
	Stay in place once attached but not forcefully adhere to the wound surface or to the periwound skin to allow easy removal and prevent stripping damage		Cost-effective material components, construction, and manufacturing process
	Minimum change of mechanical properties when exposed to body and wound fluids (endurance)		Be easy to apply and discard, for example, through easy release from the package
	Not change mechanical properties when contacting other topical therapeutic agents		Desirably include indicators for when the dressing needs to be changed or removed as it approaches its capacity to manage exudate or due to other factors that affect the life cycle
	Minimize frictional forces and shear stresses that potentially apply at the wound region to reduce potential wound tissue distortions		

Therefore, once the clinical requirements for wound care and management are defined for a given wound type and etiology, it should be theoretically possible to translate the clinically relevant characteristics of wound dressings into a physical design with specific metrics of materials, mechanical, and fluid transport and handling properties that are all determined to meet the clinical objectives and that are measurable through standardized bench testing.

This multidisciplinary critical review article, written by an International Wound Dressing Technology Expert Panel (IWDTEP), discusses the translation of clinical wound care and management into the requirements from an effective wound dressing and how such clinical requirements should determine the types, behavior, and properties of dressing materials to achieve the desirable dressing performances, as well as the need for clinically relevant laboratory test methods.

The IWDTEP appreciates that the translation of clinical wound care and management to the engineering requirements from effective dressings is a massively broad theme, even if being restricted to foam dressings. Accordingly, a long-term series of comprehensive scientific publications are planned, with the current work being the cornerstone and providing context and structure to the planned series of works.

## THE CONDENSED HISTORY OF WOUND DRESSINGS

Wound dressings are most likely the oldest type of medical device. In the ancient world and medieval times, mud, salts, feathers, leaves, cobwebs, various plant extracts, honey, and lint were commonly used to cover wounds.^4–6^ The discovery of microorganisms changed the design and manufacturing of wound care products. In the late 19th century, the Johnson & Johnson company began producing sterile surgical dressings (made of cotton and gauze) using dry-heating and pressurized steam.^7^

Further advances were made during World War I, when the first nonadherent dressings, which consisted of fabrics impregnated with soft paraffin oil, appeared to solve the problem of cotton and gauze dressings adhering to wounds, resulting in very painful dressing changes. These nonadherent dressings then evolved into two-layered dressings, a cotton fabric facing the wound that contained the paraffin and a balsam for nonadherence, and a second gauze layer on top to allow drainage by absorbency, introducing the concept of a multilayer dressing for the first time.^8^

During World War II, purified, whitened, bleached woven gauzes were developed for a variety of wound care applications and these are still used today, however, the advent of flexible polyurethane (PU) foams and the development of their mass production methods in the 1950s had again revolutionized the world of wound dressings.

Foams can absorb fluids, are vapor permeable, but retain moisture; provide mechanical cushioning and thermal insulation; and are easy to apply and remove; all of which are necessary to support effective wound healing. Altogether, these properties have progressively made foams a core dressing technology in modern wound care.

As of the 1980s, the single-foam design evolved into multilayer foam dressings based on the aforementioned historical concept of nonadherence through use of multiple layers in the dressing structure. Furthermore, other modern materials, particularly silicone, were added to the foam dressing structure, to form encased (also known as “bordered”) foam dressings, which are dressings comprising a wound contact foam pad surrounded by an adherent silicone rim.^9–11^

Today, many advanced dressings have silicone coating across their entire contact area to prevent dressing adherence, thereby minimizing the risk of damage to the wound and periwound skin upon removal. Although other advanced dressing types have been developed during the recent decades, such as superabsorbent, hydrocolloid-based, and hydrogel-based dressings, foam dressings remain a common choice for many wound care applications, and are therefore the focus of this review.

## THE PRIMARY CLINICAL ROLES OF DRESSINGS AND THEIR RELATIONSHIP AND CONTRIBUTION TO THE PROCESS OF WOUND HEALING

### The concept of moist wound healing

Odland^12^ observed that the skin epithelializes faster under unbroken blisters compared with beneath broken ones. Just a few years later, Winter^13^ presented his theory of moist wound healing, which was based on the observations of a porcine wound healing model. Winter's seminal work, which Hinman and Maibach^14^ subsequently confirmed in humans, indicated that maintaining a moist wound environment was conducive to healing, which is the concept still used today.^†^

The advantages of a moist *versus* a dry wound bed environment include the following: prevention of tissue dehydration and cell death, faster epidermal cell migration, accelerated angiogenesis, increased breakdown of dead tissues and fibrin, potentiation of growth factor interactions with their target cells, and reduction of pain.^16^ Interestingly, all of these factors are associated with the benefits of the foam dressing technology, so that the introduction of semiocclusive foam dressings (which keep the wound bed moist) and the development of the moist wound healing paradigm cross-fertilized each other.

### Clinical performance categories for wound dressings

From a clinical perspective, the primary roles of a wound dressing are to address the symptoms of the wound, that is, (i) manage exudate and, in addition, provide (ii) mechanical and (iii) biological protection to the wound, thereby substituting for the lack of a native functional skin.^17^ Any existing or new dressing can be evaluated with respect to the above fundamental three clinical requirements, based upon more specific performance categories that are listed in Table 1.

Each of the factors listed under the fundamental requirements in Table 1 is essential to the healing process and deviation from these requirements may delay, reverse, or otherwise harm the healing. For example, the fluid handling characteristics all relate to the removal of excess exudate so that the wound bed remains moist, but not wet, at all times.

Failure of a dressing to absorb the wound fluids and retain these fluids effectively under the influence of expected (*e.g.*, gravity or weight-bearing, or compression therapy) or unexpected (*e.g.*, unintentional contact with objects) *mechanical* forces, or ineffective fluid release into the environment through evaporation, may lead to backflow (reflux) of fluids into the wound area. Such wetting of the wound (potentially, with aggressive or infected exudate fluids) may cause inflammation and maceration, which are, of course, not conducive to healing.

From a *biological* perspective, any adverse response of the wound or periwound tissues to the dressing materials, or failure to prevent infection of the wound by pathogens, consumes the (already limited) inflammatory and healing resources from the wound and invests them elsewhere, for example, in an allergen response or in attacking invading bacteria. Specifically, an adverse reaction of the wound/periwound tissues to the dressing materials may provoke an excessive inflammatory response.

In theory, this should be detected before the dressings being placed on the market according to the ISO-10993-1^18^ testing standard (“Biological evaluation of medical devices”). Another cause of excessive inflammation may be the invading pathogens, which are the primary cause of chronicity and nonhealing.^‡^^,^^19,20^

## CLINICALLY RELEVANT CHARACTERISTICS OF WOUND DRESSINGS RELATE TO THEIR PHYSICAL (MICRO-)STRUCTURE

All the three fundamental requirements listed in Table 1 strongly relate to the structure of the dressing and more specifically, to the microstructural architecture and features of its material components (Fig. 1a).

Wound fluids flow into a dressing structure due to one or a combination of the following factors: (i) capillary bed pressure from the wound, which pushes the fluid into the wound cavity and from there into the dressing; (ii) gravity, which causes the mass of the fluid to be transported into the dressing; and (iii) capillary action (sorptivity), which transfers the fluid, possibly against the direction of the gravity vector, from the wound into the dressing, due to intermolecular forces between the fluid and the surrounding solid surfaces.^21^

Once the fluid has penetrated the dressing structure, the fluid handling and the intrinsic absorption capacity of an applied foam dressing are facilitated by the microporous structure of the foam (Fig. 1b). The porosity of the foam and the level of interconnectivity between the micropores in the foam are very likely to influence the fluid transport properties of a foam dressing.

However, it is important to note that the existing laboratory test methods, including those specified in the European test standard 13726-1,^22^ are not designed to capture the time course of fluid transport. Therefore, there is a clear need to include new test methods in the future, improved testing standards, to measure and record the performance of wound dressings as they are used clinically, over a time course, during the indicated period of use.

### The microstructure of foam dressings and the potential effects on performance

Absorbency in foam dressings is achieved by transport of exudate into the open cells of the foam structure, through interconnecting channels, and hence, the absorbency capacity of foam dressings is determined largely by the porosity of their foams (*i.e.*, the volume fraction of the micropores). The fluid handling characteristics of foam dressings are constituted by the interaction of this absorbency capacity with the moisture-vapor transmission rate (MVTR) that is determined by the permeability of the backing material/film. That is, after the initial fluid uptake into the foam structure *via* absorbency, evaporation of the fluid through the backing material/film largely controls the fluid management of the dressing.

The MVTR keeps the dressing from becoming saturated, and an adequate MVTR level of the dressing will maintain the wound bed moisture through the use period. As evaporation takes place through the backing material/film, additional moisture can be removed from the wound through absorption into the foam. Accordingly, through evaporation, the total amount of moisture removed from a wound can exceed the absorbency capacity of the foam. More permeable backing materials/films permit higher evaporation rates from the dressing, thus providing the potential for longer wear times.

On the contrary, if the MVTR is set too low in the design of the dressing, then at a certain time during usage, newly inflowing exudate arriving from the wound will not have space in the micropores, resulting in leakage from the dressing and maceration.

Of note, unlike superabsorbent wound dressings that actively retain and “lock in” fluid through hydrogen bonding with water molecules, foams hold in the exudate passively in their voids, and hence, will release fluid under the influence of mechanical forces such as bodyweight or external forces that substantially deform the dressing. In other words, deformations of the dressing by bodyweight or external forces decrease the size of the micropores, squeezing fluid out of the dressing (as in a squashed sponge), which may compromise the wound or periwound.

Foams used in commercial wound dressings have variable pore sizes, ranging from 25 μm to over 1,000 μm.^23^ Smaller pore sizes and interconnecting channels allow for greater fluid retention properties. Larger pore sizes typically facilitate increased fluid absorption from the wound into the dressing, better handling of viscous fluids, and greater evaporation from the dressing to the environment.^24^

The downside though is that larger pores and connecting channels allow immune and tissue-repairing cells such as fibroblasts to migrate into the dressing structure, instead of remaining within the wound bed,^25^ which in turn impairs healing. Furthermore, fibroblasts infiltrating the porous dressing structure may synthesize collagen within the dressing, near the wound surface, which would effectively lead to connective tissue (micro-)ingrowth into the dressing, resulting in traumatic and painful dressing removals.

Even if the pore sizes are not large enough for cells to infiltrate into the dressing, they may be sufficiently large to facilitate fibrin deposition at the wound contacting layer (as fibrin is present in the exudate at the early healing stages). The deposited fibrin may then cause dressing adherence (acting like a fibrin glue), especially if the dressing was *in situ* for multiple days.^26^

Many of these historical problems related to tissue ingrowth into dressings and fibrin deposition, resulting in dressing adherence (leading to tissue trauma and pain to the patient on dressing removals), have been addressed by the introduction of dressings with silicone at the skin and wound interfaces.^27–33^ Yet, discomfort and pain during dressing removals remain a clinical issue, for example, because for dressings in which soft silicones are used to provide the adhesive border only, the wound contact layer (not covered by the silicone) might adhere to some degree to the wound bed.^32^

Accordingly, the above considerations highlight the complexity of choosing the correct porosity and connectivity for the microstructures of foams in wound dressings. Given the existing wide variety of porosities in foam dressing products,^23^ what is an ideal porosity that perfectly balances between absorbency, retention, and occlusion of tissue ingrowth into the dressing, or fibrin deposition, obviously remains an open question.

Further complications arise from the fact that, for foam dressings, porosity and connectivity affect their mechanical (strength and flexibility) characteristics. For example, smaller pores indicate greater density of the dressing and, correspondingly, a stiffer material behavior.^34,35^ A foam dressing that is excessively stiff may indent the skin and cause deformation-inflicted tissue damage.^36^

**Figure 3.**
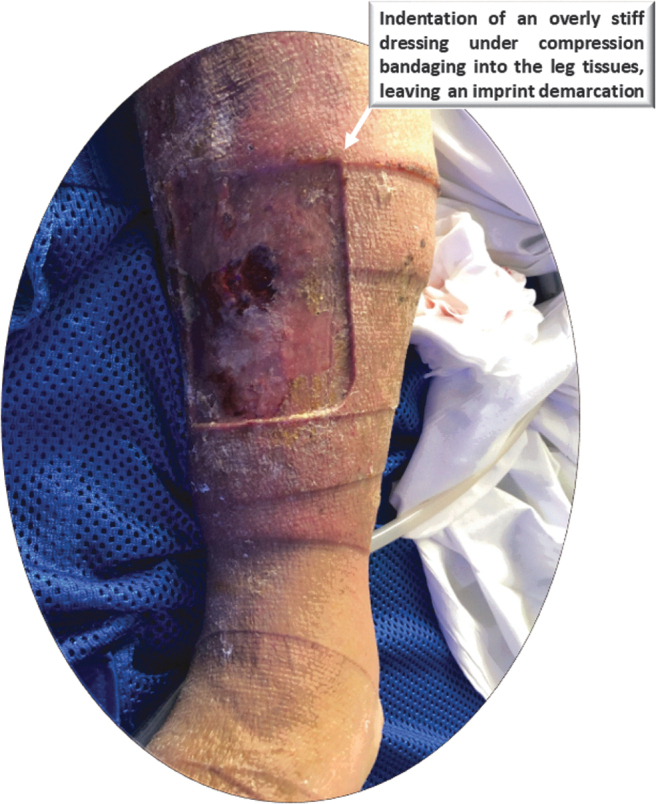
A clinical documentation of the effect of an excessively stiff foam dressing on the periwound skin. The image shows the wound of a 67-year-old man with a history of obesity and chronic leg ulcers due to venous lymphedema. This patient developed a pretibial ulcer with moderate amount of drainage. A rectangular foam dressing with sharp corners (*white arrow* marking) was applied under a two-layer compression therapy system for at least five consecutive days. The deep indentation on the periwound skin, which was also associated with pain, is clearly visible. Of note, the dressing may not have been appropriately chosen by the clinician caring for this wound. The documentation of this case is courtesy of author K.W.

A clinical case demonstrating this phenomenon is shown in Fig. 3, which documents a wound of a 67-year-old man with a history of obesity and chronic leg ulcers due to venous lymphedema. This patient developed a pretibial ulcer with a moderate amount of drainage. A rectangular foam dressing was applied under compression therapy that was changed every 5 to 7 days. Not only was the choice of the foam dressing material too stiff in this case, but the dressing was also applied under a two-layer compression system, which caused an “imprint” of this overly stiff dressing into the skin under the compressive forces.

The selection of the dressing, with its sharp corners that induced mechanical stress concentrations in skin and underlying soft tissues^§^ (Fig. 3; white arrow marking), indicates that this was not an optimal compression treatment, and the dressing itself may not have been appropriately chosen by the clinician caring for this wound. Indeed, this dressing left a deep, well-demarcated indentation on the periwound skin, which was also associated with pain (Fig. 3). The patient case presented in Fig. 3 exemplifies how an inadequate engineering dressing property (excessive material stiffness in this case) and the dressing geometry with its sharp corners may lead to an adverse event and failure of the dressing in clinical practice.

In contrast to the above, larger pores (*i.e.*, lower density of the foam) mean more entrapped air and less solid polymer phase, resulting in lower strength and stiffness of the foam.^34,35^ While low stiffness is theoretically beneficial for a wound dressing, as it minimizes abrasion of the wound or indentation of the dressing into the periwound skin (Fig. 3), the associated decreased strength may, in theory, allow occasional low-intensity forces to damage and degrade the dressing microstructure (Table 1).

Consideration of the strength, stiffness, and fluid transport and handling properties for each material component in the design optimization of multilayer foam dressings (as relevant to their clinical performance) greatly increases the complexity of the dressing's structural optimization task.

### Permeability and breathability of the external dressing surfaces

The design of an adequate backing material/film of a dressing can be similarly considered an outcome of an engineering optimization problem. On the one hand, the backing material/film should have sufficient permeability to achieve an adequate MVTR, so that fluids are constantly removed from the dressing reservoir by evaporation to the environment (thereby making space for additional exudate, as explained above) (Table 1).^38^

On the other hand, the backing material/film should have a sufficiently low permeability to prevent pathogens and other irritants from entering the wound (and from there, into the circulation) (Table 1). Furthermore, the MVTR is affected by the ambient temperature, relative humidity, and air velocity at the vicinity of the applied dressing, as well as by any additional coverage of the dressing, such as by clothing or bedsheets.^39^

Once again, the ideal permeability for the backing materials/films of wound dressings is currently unknown,^40^ although some manufacturers introduced a “moisture control layer” in their foam dressings to modulate the MVTR through the backing material/film under varying wound conditions.^38^

Of note, foam dressings may also include a soft, porated silicone sheet over the wound contact layer, to allow absorbency of exudates into the dressing, in which case the pore size and density in the silicone sheet will affect both the rate and the amount of the absorbed and retained exudates (also, while some bacteria are known to adhere to silicones, that can be minimized through surface treatments/preconditioning and/or by embedding silver ions in the foam layer).

## EXUDATE MANAGEMENT AND DRESSING FUNCTION ACROSS THE RANGE OF ACUTE AND CHRONIC WOUNDS

The most relevant physical law governing the mode of action of all foam dressings is Darcy's Law. This physical law, which generally applies to relatively slow-moving flows, determines that the rate of the exudate flow into a foam dressing is inversely proportional to the viscosity of the inflowing exudate.^41,42^ According to Darcy's law, aqueous exudates with a relatively low viscosity flow into a dressing faster than more viscous exudates managed by the same dressing.

Therefore, the effective permeability of the dressing structure, which is influenced by the density, porosity, and interconnectivity of the micropores in the foams that make up each layer of the dressing, should be considered in the context of the exudate viscosity, and is the key performance parameter that ultimately determines absorbency.

If the permeability of any of the layers in the dressing to an exudate of certain viscosity (typically high viscosity) is insufficient, more viscous exudates will not be able to effectively penetrate the inner dressing layers and the dressing will therefore not absorb and retain a sufficient amount of wound fluids. In such cases, exudate pooling may occur or the exudate might flow back into the wound area and accumulate, causing further inflammation and/or maceration, thereby potentially increasing the wound size.

In addition, if the exudate contains solid particles such as blood clots, or aggregates of dead cells or proteins, the dressing could act as a filter, absorbing the fluid components from the exudate but leaving the solid particles at the wound bed, where they would (similarly to excess fluid) compromise the healing.

**Figure 4.**
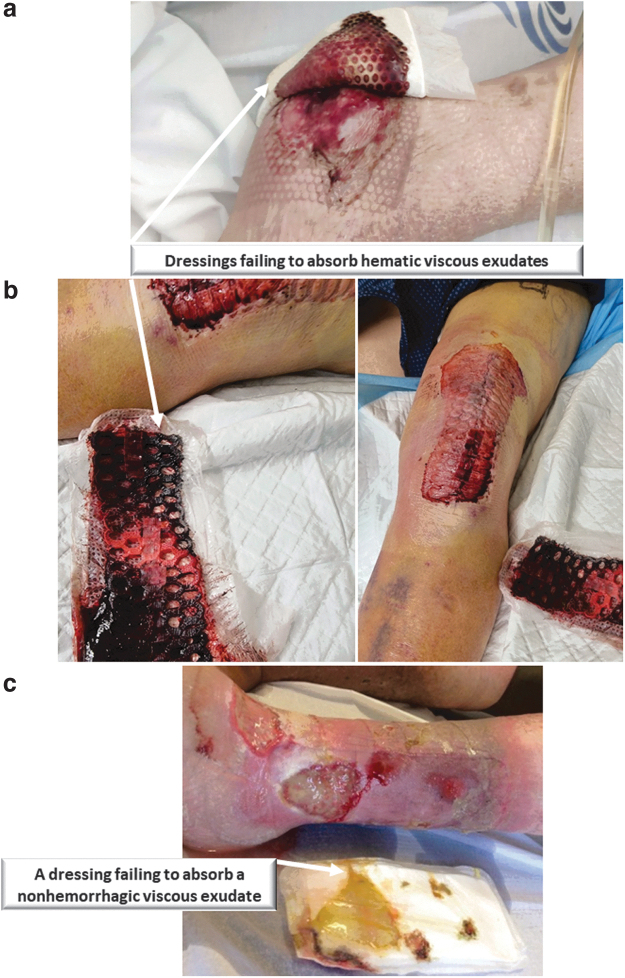
Clinical documentations of dressing failures due to poor absorbency of viscous fluids in the care of acute and chronic wounds: **(a)** A highly exuding heel pressure ulcer where the polyurethane foam dressing had failed to absorb the viscous hematic exudate. The documentation of this case is courtesy of author PA. **(b)** A 60+ year-old male patient who underwent a left knee repair was at risk of postoperative bleeding. A conventional postoperative dressing was applied, but clearly failed (the images were taken at day 4 postsurgery), because the dressing needed to handle a relatively large amount of whole blood, which is considerably more viscous than plasma exudation. While appreciating that ideally, hemostasis of the wound should be achieved before applying a dressing, and therefore, bleeding into a dressing presents a considerable challenge for its fluid management and handling performance, in these two cases shown here, the absorption rate into the dressing was too slow and the rate of the incoming blood flow was too high, resulting in pooling of blood under the dressing (as opposed to the absorption and retention that a dressing is expected to achieve). The documentation of this case is courtesy of author TS. **(c)** An 82-year-old male patient with multiple venous leg ulcers on the same leg that release exudates with different viscosities. The dressing was clearly unable to absorb the more viscous exudate. The documentation of this case is courtesy of author P.A.

Clinical cases illustrating this type of dressing failure in acute care are documented in Fig. 4a and b. The first image (Fig. 4a) is of a wound of a 73-year-old female poststroke with left hemiparesis and impaired mobility and sensibility, who also suffered depression and malnourishment, where the applied PU foam dressing had failed to absorb the viscous hematic wound fluids from her highly exuding heel pressure ulcer.

Similarly, in the second illustrative case (Fig. 4b), a male patient older than 60 years underwent a left knee repair procedure and was at risk of postoperative bleeding. A conventional postoperative dressing was applied in the latter case, but it failed because the dressing had to handle a relatively large amount of whole blood, which is substantially more viscous than plasma exudation (by approximately fivefold^21^).

While appreciating that ideally, hemostasis of the wound should be achieved before applying a dressing, and therefore, bleeding into a dressing presents a considerable challenge for its fluid management and handling performance, in the above two documented cases, the rate of absorption into the dressing was too slow and the rate of the incoming blood flow was too high, resulting in an accumulation of blood under the dressing.

This stands in contrast to the fundamental clinical requirement that a dressing should achieve absorption and retention in all cases—both for aqueous and viscous exudates. Another similar clinical case of a dressing that had failed to absorb a viscous exudate, this time in the context of care of chronic wounds, is shown in Fig. 4c. This latter case is an 82-year-old male patient with multiple venous leg ulcers on the same leg that released exudates with different viscosities. The applied dressing clearly failed to absorb the more viscous discharge.

All of these clinical cases of dressing failures described above, together with the human factors that were involved in each case (Figs. 3 and 4), led to medical device-related adverse events. Unfortunately, however, in clinical practice, such dressing failure events are often left unreported or are considered minor issues, which prevents or delays regulatory actions (contrarily, for example, to problems observed with implantable devices that are promptly reported to regulatory bodies through a Medical Device Reporting process).

## THE REQUIRED DRESSING-WOUND/SKIN INTERFACE CONDITIONS DURING THE USE AND REMOVAL OF DRESSINGS

An adequately performing wound dressing must “stay in place” over the periwound skin during use, even in a moist environment, but should also allow for easy, atraumatic, painless, and as convenient as possible removal when a change is required.^43^ Adhesive dressings may strip layers of the periwound skin.^44^ This causes discomfort or pain during removals and further compromises the integrity of the skin (particularly the stratum corneum), adversely affects transepidermal water loss, and promotes inflammation.^45–47^ The above processes are repetitive, as skin damage accumulates each time a dressing is removed from the wound.^48^

In people with sensitive skin (*e.g.*, the elderly or infants), excessive adhesion of a dressing to the skin near the wound can cause skin tears.^49^ In addition, painful episodes during dressing changes can cause chronic psychological stress in patients, which in itself delays wound healing.^50^ The requirements of “stay in place” and “no skin peeling” are seemingly contradictive and again require careful optimization of wound dressing designs, which is another bioengineering challenge.

From a patient's perspective, a dressing that is not adhesive enough may slip and move during wear, hindering its ability to manage exudate and not facilitating showering for the user. Since chronic wounds are typically present for many weeks or months, the ability of a person to shower regularly with the dressing remaining in place is also a necessary property for their quality of life. Related to that, and from an engineering testing point of view, the adhesive strength of a dressing onto a skin-mimicking material can be measured using a laboratory “peel test,” which is designed to determine the adhesive bond strength or the tacky property between the dressing and a (dry or wet) skin simulant.

Nevertheless, despite decades of research, no suitable material to optimally mimic skin has been found that would allow for reproducible adhesion force testing and determination of which forces would be considered being comparable with what native skin is exposed to clinically when dressings are removed. Traditionally, glass and steel plates are therefore commonly used as a substrate for peel tests; for reproducibility, however, efforts are underway to develop more biomimetic skin substitutes for this purpose, for example, based on blends of natural proteins in gelatin, to better represent the interactions occurring at the skin/adhesive interface with respect to conventional substrates for peel tests.^51^

In physical terms, laboratory peel tests measure the resistance forces to separation of the dressing from the skin substitute (or in some cases, on the skin of study subjects), to determine if these forces are sufficient or perhaps excessively high.^52^ To control these separation forces, engineers design textured adhesive surfaces so that only a portion of the surface (with the higher surface topography) makes contact with the skin. A relevant test standard for these measurements is ASTM D903-98.^53^ Of note, the specific substrate to measure against is not stipulated in the aforementioned test standard, despite the choice of the substrate being important for determining the clinical relevance of the method, as indicated above.

There are several other problems associated with laboratory evaluations of dressing removals. For example, it is extremely difficult to correlate measured force values with expected levels of discomfort or pain, because discomfort/pain is always subjective and influenced by numerous patient-specific conditions such as the nature of the wound, medications, sedation, hyperalgesia, allodynia, and neuropathy, as well as the anatomical location of the wound (as some body areas are more sensitive than others).

Second, the technique of removing a dressing has a strong effect on the measured separation forces, as both skin simulants and dressing materials are viscoelastic and the state of contact stresses between the dressing and the skin therefore depends on the speed of removal.

Once a health care professional begins to remove a dressing from the skin, the contact area between the skin and the dressing continues to decrease until the dressing completely detaches from the skin. Since the amount of mechanical stress on the skin at the wound site depends on the ratio between the separation (removal) force and the (continuously decreasing) contact area, a clinician would need to lower the removal force in proportion to the remaining contact area between the skin and the dressing, to avoid applying too much mechanical stress on the skin at the wound site, but this is difficult to control manually and requires skill and experience.

Moreover, appreciation and knowledge of how to apply such bioengineering principles to the bedside are lacking, and are not commonly taught.

In addition, the viscoelasticity of the skin and dressing will increase the stresses on skin at the wound site with an increased rate of peeling, which is the scientific rationale for the medical recommendation to remove dressings slowly.^49,54^ All of these real-world factors that point to the influence of dressing removal techniques (in terms of clinical skills), as well as to the skin health status, are not considered in the current laboratory work. Similar to the other aspects discussed above, more clinically relevant testing standards that consider real-world conditions should be developed to represent the real-life factors related to wound dressing removals in laboratory performance evaluations.

## THE HOSTILE BIOPHYSIOLOGICAL ENVIRONMENTS WHERE DRESSINGS MUST FUNCTION AND THEIR POTENTIAL IMPACT ON DRESSING PERFORMANCES

### The pH of wound exudates and their potential influence on foam dressings

The biochemical state of a wound, and in particular, the pH level in the wound bed, has been shown to have a significant impact on various aspects of the healing cascade including cell proliferation and migration, the proteolytic environment including the metalloproteinase enzyme levels, angiogenesis, and antimicrobial activity.^55–57^ Depending on its material composition and the level of occlusion provided, a dressing can alter the pH of the wound,^58^ and should remain structurally and functionally tolerant to highly acidic or alkaline wounds.

For example, the pH of exudates in burns ranges from 5 (which is in the middle range of normal skin pH) to as high as 10, and such elevated pH values are typically associated with local infections.^59^ Combined with mechanical forces that occasionally act on a dressing and the enzymatic agents in wound exudates, extreme alkaline pH values can challenge the chemical resistance of some PU foams, thereby leading to the release of dressing materials into the wound or a breakdown in the structure of the dressing.^60,61^

### Cell biology and microbiology aspects

Another important and relevant consideration is that the presence of a wound dressing may interact with the coordinated and combined efforts of the cells involved in the wound healing process, for example, keratinocytes, fibroblasts, endothelial cells, neutrophils, and macrophages. The migration, infiltration, proliferation, and differentiation of these cell types are triggered and regulated by complex molecular signaling involving growth factors, cytokines, and chemokines.

Numerous research articles and textbooks were published concerning the involvement of specific growth factors in these coordinated efforts, such as the fibroblast growth factor, epidermal growth factor, transforming growth factor-beta, connective tissue growth factor, vascular endothelial growth factor, granulocyte macrophage colony stimulating factor, platelet-derived growth factor, and tumor necrosis factor-alpha families, to mention a few, as reviewed by Field and Kerstein^16^ and the references cited therein.

Likewise, members of the interleukin family of cytokines are known to be involved in controlling the intensity of the inflammatory process in the wound and its surroundings, through signaling between cells of the same phenotype and across different cell groups. Accordingly, the materials of the dressing must not affect the molecular conformation, intermolecular interactions, or chemical stability of any of these proteins.

The presence of infection is another critical biological aspect, affecting the clinical management of wound care. Foam dressings can also be manufactured to release antibacterial and antifungal agents such as silver ions for antimicrobial protection, as wound infections, particularly in combination with neuropathy and/or ischemia, for example, as in a diabetic foot ulcer, may lead to gangrenes and amputations. As potential leakage of infected exudate from a dressing may transfer the infection to other body regions, using silver-containing foams, in addition to frequent dressing changes, can also mitigate the risk of spread of the infection beyond the existing wound.

In this context, Davies *et al.* recently reported the results of a global electronic survey in which they found that dressing changes are typically performed 1–2 days earlier in infected wounds than in noninfected wounds with a corresponding etiology.^62^ The more frequent dressing changes for the infected wounds may be associated with either the need for change of a wet dressing, or because the clinician is more concerned about an infected wound and would want the dressing changed more frequently (many dressing manufacturer's recommend daily dressing changes for infected wounds, due to the greater risk associated with infected wounds and the need to ensure that the wound is not deteriorating).

Regardless of the clinical reasoning for the frequent dressing changes, the effects of the presence of microorganisms on the structure and function of a dressing must be taken into account in engineering design endeavors and bioengineering laboratory evaluations of existing and new wound dressings.

#### The quality of attachment of the dressing to the wound site

Another important challenge in a wound dressing design is premature detachment due to moisture from the wound exudate or perspiration in the wound environment, which compromises the adhesive/tacky function of the wound-facing aspect of the dressing.^63^ Premature detachment of wound dressings is common and can occur as early as the day after application of the dressing, with the occurrence increasing over the next few days (up to day 5, which was the endpoint of the work by Rippon *et al.*^63^).

Global surveys of nurses found that a dressing is changed at least once every ∼3 days if the wound is infected, or at most every ∼6 days if the wound is not infected, regardless of the wound etiology.^62^ Therefore, if the design of the dressing is such that body fluids are trapped at the edges of the dressing and cannot effectively escape by evaporation, the dressing will detach earlier than expected by the wound care professional (as documented in the work of Rippon *et al.*^63^).

In other words, the absorptive and retentive properties of a foam dressing are also key to reducing the risk of premature detachment of dressings. Such premature dressing detachments not only cause additional health care labor and costs associated with the more frequent than necessary dressing changes, but also jeopardize the goal of undisturbed healing, which is a consistent requirement in contemporary wound care practice.^43,64^

## ADDITIONAL IDENTIFIED GAPS IN THE EVALUATION AND TESTING OF WOUND DRESSING PERFORMANCE WITH RESPECT TO CLINICAL NEEDS

### Evaluating pain and discomfort during wound care procedures

Pain and discomfort during common wound care procedures are considered a primary patient-centered outcome, particularly in the context of dressing changes, which are often a painful experience for patients. Gardner *et al.* reported that dressing changes caused considerable pain in 74% of the patients in their study, half of whom defined that pain as being severe.^65^

While many of the historical problems of tissue ingrowth into dressings and dressing adherence (leading to tissue trauma and pain to the patient on dressing removal) have been addressed by the introduction of foam dressings with silicone wound/skin interfaces,^27,66^ pain and discomfort during dressing removals continue to be a source of stress and anxiety for patients, who often mention these issues as a major quality-of-life indicator.^50,67^

Price *et al.*^68^ conducted a survey among 2018 patients with wounds across 15 countries, and found that ∼40% of their study participants indicated that the pain at dressing change was the worst part of living with a wound.

Importantly, there is a strong association between the pain and discomfort during such dressing changes and poor exudate management performance of the relevant dressing (*e.g.*, as demonstrated in Fig. 4). For example, excess exudate that dries on the periwound skin or the dressing may increase the pain sensation during removals of the chosen dressing type.

Some recent studies used heart rate variability (HRV) analyses of electrocardiographic measurements to quantify pain and discomfort during dressing changes. This had been based on literature demonstrating that pain episodes are associated with a decrease in the power spectral analysis of the HRV, which serves as a measure of the activities of the sympathetic and parasympathetic components of the autonomic nervous system.^69^

Using an HRV-based method, Razjouyan *et al.* reported that high-intensity pain causes substantial mental stress for patients that may negatively affect their wound healing outcomes.^70^ Clearly, the selection of a dressing type, its materials, structure, and composition, has an important role in potentially reducing the discomfort and pain during dressing changes, which in turn, influences the healing rate.^70^

### Gaps between laboratory fluid handling tests and real-world exudate management

Another common problem in evaluating the performance of wound dressings with respect to the mechanism of failure, shown in Fig. 4, is that laboratory tests conducted by industry and academia often consider only the fluid management properties of wound dressings tested by exposure to saline, Ringer's solution, or similar solutions prepared by dissolving salts in water.^71^ In the European standard 13726-1,^22^ the test fluid is specified as “Solution A,” a water solution of sodium chloride and calcium chloride. Exudates in the real world contain proteins, ions, and cells that make them substantially more heterogeneous and viscous than water.^21,71–73^

Therefore, a dressing that exhibits adequate absorbency and retention capabilities in a laboratory test performed with such aqueous solutions may fail in a clinical scenario where it must manage viscous fluids that barely penetrate the first layer of the dressing facing the wound and hardly ever reach the core of the dressing, effectively disabling the absorbency and retention features of this failing dressing (Fig. 4).

Furthermore, multilayer foam dressings evaluated in laboratory studies are often subjected to soaking tests, such as in the absorbency tests described in the European test standard 13726-1^22^ for primary wound dressings (critically reviewed by Gefen and Santamaria^74^). Such soaking tests ignore the basic mode of action of multilayer foam dressings, where fluid penetration and absorption into the different dressing layers progress from the layer in contact with the wound to the deeper layers of the dressing. This progression of fluid does not necessarily occur at equal rates across the layers of the dressing.

When a dressing is soaked in fluid and brought to a fully saturated state, no information can be obtained about the extent of absorbency in each of the individual dressing layers, and how the fluid has progressed within the dressing over time.^74^ Importantly, the types of dressing failures documented in Fig. 4 cannot be captured in a soaking test because if fluid can penetrate the dressing from all sides, the information concerning the clinically relevant time course of the fluid penetration into the dressing, and of the transport process within the dressing, is lost completely. This means that a dressing may “pass” the aforementioned 13726-1 test, but fail clinically.

## SUMMARY AND CONCLUDING REMARKS

The objective of this review article is to describe the interplay between the structure and function of wound dressings and identify relevant gaps in knowledge. More specifically, we demonstrated that multilayer (or even single-layer) foam dressings are not all created equal, but rather, their performance is based on their specific material composition and construction. To be clinically effective, a foam dressing must be able to handle a wide range of exudate viscosities associated with different wound etiologies, or with the same wound at different stages of healing.

Current industry test standards often use aqueous solutions (with different salinity levels), without proteins to test dressing performance, which is unrealistic and oversimplifies the complexity of the clinical demands of real-world wounds. There are a few examples for academic research and companies reporting the use of protein containing test liquids^75,76^ and more viscous test fluids.^21,77–80^ However, since these test solutions are not included in the EN 13726-1^22^ standard, the tests are often not eligible for consideration in hospital formularies.

Bioengineering studies in the area of dressing efficacy research are therefore essential to advance this field, for example, by measuring the ranges of exudate viscosities for different wound etiologies and stages of healing toward establishing standards for test fluids. Ultimately, the industry would need to develop a range of exudate substitute fluids capable of representing the existing diversity of native biological exudates, to enable clinically relevant laboratory testing and the formulation of new testing standards for wound dressings. This work is ongoing in the first author's (A.G.) research group.^21,74,80^

Finding the ideal balance between feasible, robust, and reproducible laboratory testing of wound dressings *versus* the need to reflect the vast clinical complexity in such engineering tests will continue to be a challenge for bioengineers. The variety of wound etiologies, patient characteristics, and underlying diseases requires that bioengineers in academia and industry work closely with wound care clinicians to develop realistic yet practical bench tests that would optimize the quality and reproducibility of these tests with respect to the clinical practice.

The IWDTEP recommends that for foam dressings, such bench tests will be designed to quantitatively acquire the basic engineering performance metrics detailed in Table 2, based on mapping the gaps between existing testing standards for wound dressings and real-world clinical conditions, as listed in Table 3. This table lists the fundamental and quantifiable parameters (through engineering laboratory testing) as a core conceptual framework for the design and evaluation of existing and new foam dressings.

**Table 2.** Description of the basic engineering performance metrics required from a foam dressing that can be quantitatively measured in laboratory settings

Fluid Handling		Mechanical		Biological	
Manage a range of viscosities	The dressing should absorb and retain exudate effectively and consistently in terms of volume and mass over the clinically relevant time course, across the entire reported range of possible human biofluid viscosities, without the occurrence of spillovers or pooling	Stiffness	The dressing stiffness should not substantially exceed the skin and subcutaneous tissue stiffnesses relevant to the location, etiology, and severity of the wound, at either its new (straight-from-the-package) condition, or post a usage period, to avoid indentation damage	Permeability to pathogens	The dressing should prevent penetration or release of particulates at sizes representative of bacterial, viral, and fungal pathogens through the external dressing surface
Body position, wound location, and interaction with additional wound care treatments	The dressing should absorb and retain exudate effectively and consistently across all the possible positions of the body and wound with respect to the gravity vector, for example, when the dressing is vertical to the ground (such as when treating a venous leg ulcer), or when the dressing is subjected to sustained or repeated bodyweight forces (as in a nonoff-loaded wound). Addition of external compressive forces, such as during application of multilayer compression bandaging and other forms of leg compression treatments applied for the management of venous insufficiency or venous leg ulcers should minimally affect the absorbency and retention performance of the applied dressing	Strength	The dressing should be durable to the expected compressive, tensile, and shear force magnitudes associated with the bodyweight, including any potential body movements against surfaces (in single or repeated events), and to the pull-out forces when the used dressing is removed, at its new (straight-from-the-package; also considering storage duration and conditions), or post a usage period		
Consistent fluid evaporation to the environment	The dressing should constantly and consistently release the retained fluid to the environment, *via* evaporation, for the ranges of clinically relevant temperatures and humidity values representing the ambient conditions at the potential or the intended geographical regions of use (*e.g.*, in an extremely moist or an extremely dry environment)	Adhesiveness	The borders of the dressing should provide consistent adhesiveness throughout the intended period of use, that is, the dressing should stay in place under the expected regimens of the bodyweight and external forces, as well as under the influence of skin moisture conditions, but should not require excessive peeling forces during removal to avoid periwound skin stripping damage. Contrarily, the wound pad should never adhere to the wound bed (*i.e.*, for the wound pad, measured peeling forces should be extremely low)		

**Table 3.** Important gaps between existing testing standards for wound dressings and real-world clinical conditions

Fluid Handling		Mechanical	
Test Standard	Major Weaknesses	Test Standard	Major Weaknesses
EN 13726-1 Test methods for primary wound dressings—Part 1: *Aspects of absorbency* (specifies laboratory test methods recommended for the evaluation of absorbency of primary wound dressings)	• Does not consider physiological directional flows (from the wound-bed into the wound pad), as it utilizes free-swell measures for dressings submerged in excess test fluid • Does not consider the combined effects of gravity and bodyweight-induced or other forces on the flow conditions, such as the variable roles of natural convection *versus* capillary motion (depending on the specific wound and dressing orientations with respect to the ground), and any potential distortion and reduction of the available dressing reservoir for absorbency and retention by bodyweight or external forces • Does not address the protein contents and the associated range of fluid viscosities that exist for biological wound exudates (as this test utilizes an aqueous test fluid, *i.e.*, salts dissolved in water termed “Test Solution A”) for the absorbency measures specified therein	EN 13726-4 Test methods for primary wound dressings—Part 4: *Conformability* (describes a laboratory test method for measuring the conformability of primary wound dressings)	• Does not consider the real-world, clinically relevant shape conformation phenomena that involve bending and shearing, for example, of wound dressings applied to irregularly curved body regions (such as the posterior heel, the nasal bridge, or the ears), as the test is limited to tensile elasticity only • Does not consider mechanical durability (also known as “fatigue”) factors or real-world wear-and-tear phenomena, and their potential effects on the structural integrity of wound dressings • There is no consideration of the stiffness matching between the tested dressing materials and native skin (or underlying soft tissues), in the context of preventing indentation damage to the periwound skin
EN 13726-2 Test methods for primary wound dressings—Part 2: *Moisture-vapor transmission rate of permeable film dressings*	• Does not consider protein contents in the test fluid, and, as a result, neglects the possibly modified kinetics of evaporation of protein-rich fluids (as proteins may have hydrophobic regions on their surface, which in turn, may affect their evaporation kinetics)	ISO 29862:2007(en) Self-adhesive tapes—Determination of peel adhesion properties (concerns the laboratory measurements of separation forces required to peel a strip of adhesive tape from industrial steel plates)	• Does not consider the deformability of human skin, and hence is poorly relevant to either healthy or fragile skin response. Specifically, as steel is rigid (*i.e.*, not deformable and viscoelastic as native skin is), it is not representative of the complex biomechanical phenomena that occur during removal of wound dressings. For example, the level of deformability of the surfaces that are subjected to separation strongly affects the dynamic separation forces that form between the separating surfaces. A faster peeling action applied by a wound care clinician would induce a higher deformation rate of the skin, and, due to the viscoelasticity of hydrated skin that would result in greater peeling forces (which are associated with the rate of deformation). A rigid steel substrate cannot represent this complex mechanical behavior of the biphasic (solid-fluid) skin tissue • Does not consider the microtopography features of skin. Specifically, as the industrial steel plate substrates are relatively smooth, and do not contain the inherent roughness and possible wrinkling of human skin, the realistic contact area of the dressing adhesives with skin is not adequately represented, which again biases the peeling force measurements
EN 13726-3 Test methods for primary wound dressings—Part 3: *Waterproofness* (describes a laboratory test method for the evaluation of the waterproofness of primary wound dressings when such claims are made)	• Does not consider the influence of the body and wound temperatures, and thereby, the effect of the resulting (temperature-dependent) fluid surface tension changes on the waterproofness (as the test solution is used at room temperature) • Does not account for alkaline or acidic fluids where the fluid with non-neutral pH may affect the fluid repellent or interact differently with the dressing materials. This is particularly important for nonwatery, protein-rich fluids (not considered in the test but representative of the majority of biological exudates), where a rise in the pH can change the protein conformation, and thereby, the surface tension interactions		

The relevant, widely accepted international test standards are the following: (i) European Standard EN 13726 “Non-active medical devices: Test methods for primary wound dressings”; and (ii) ISO 29862:2007(en) “Self-adhesive tapes—Determination of peel adhesion properties” (which is based on, and improved from the previous EN 1939 standard).

In some of the patient cases shown (Figs. 3 and 4), the selected dressings were likely not the appropriate or optimal choice for the treated wounds, which was the root cause for the failure. Nevertheless, the examples that were provided demonstrate that foam-based or other types of dressings may be challenged in terms of fluid management, for example, by wounds with thick and viscous exudates, or prove to be overly stiff for the type of treatment (such as in compression therapy), and the failure cases could have been prevented by not selecting these specific dressings for the tasks.

Clearly, control over hemostasis is fundamental in wound care and spans beyond the dressing choice, but the failure cases that were presented, namely compromised management of highly viscous fluids and the excessive stiffness of the dressing with respect to that of the wound and periwound, are points that clinicians should be aware of, and consider when choosing a specific dressing for a specific wound and treatment approach. For example, with regard to the case shown in Fig. 3, while the key foams used in wound dressings are either PU or polyvinyl alcohol (PVA), PU foams generally exhibit more uniform pore sizes and better interconnectivity between the pores, as well as substantially less swelling, and hence, lower stiffness at their wet condition with respect to the PVA foams.^81,82^

The latter implies that PVA foam dressings should not be used under compression therapy, which again exemplifies that foam dressings may vary considerably in their clinical performance, depending on the specific foam materials and microstructures.

Pain associated with dressings may be related to adherence of the dressing to the wound bed, or to periwound skin stripping damage during removals, or be related to failure of the dressing to manage the exudate, leading to maceration due to pooling or spillover of the exudate. The depth of tissue loss may be associated with the intensity of the resulting pain, because the density of nociceptors—the neural sensory receptors that detect signals from damaged tissues—differs across tissue types (cutaneous tissues are generally more densely innervated than deeper soft tissues).

The seminal position document of the European Wound Management Association concerning pain at wound dressing changes indicated that a dressing should maintain moist wound healing to (among the other factors reviewed in our current work) reduce friction at the wound surface, and thereby lower the frictional rubbing against the wound.^83^

In addition, the dressing should also remain attached for longer times, to reduce the need for frequent dressing changes.^83^ In this regard, Alvarez *et al.* commented that compared with dressings with traditional adhesives, the use of dressings incorporating soft silicone can minimize traumatic injuries to the wound-bed and periwound skin, reduce dressing-associated trauma, and thereby, reduce the discomfort and pain.^84^

Finally, clinicians should adopt proactive critical thinking and inquire about the specifications of foam dressing technologies that are being offered to them. Laboratory test data should be requested from manufacturers, to verify that the dressing being considered is capable of handling the fluids relevant to the wound etiologies that are treated, such as the expected exudate volumes, flow rates, and viscosities.

Requiring manufacturers to agree upon and implement standardized clinically relevant test methods for their wound dressing products, and then provide peer-reviewed, published laboratory test data based on the implemented testing standards will lead to informed clinical decision-making regarding the selection of the safest and best performing foam dressings.

Such dialogue between clinicians and industry will further positively impact patient safety, quality of care, and the overall cost-effectiveness of treatments. Furthermore, it is essential for optimal care to use appropriate wound dressings that can effectively absorb exudates, are resistant to the mechanical and biochemical wound environment, remain in place for the required treatment period but can be easily removed, and are acceptable to both patients and health care professionals.

The only way for clinicians to fully trust wound dressing products is to develop clinically relevant laboratory test standards that result in comprehensive performance metrics for dressings. Such clinically relevant testing standards would allow objective, standardized, and quantitative comparisons between wound dressing products and brands, leading to informed decisions.

**TAKE-HOME MESSAGES**

- It is essential for optimal wound care to use appropriate dressings that can effectively absorb exudates, are resistant to the mechanical and biochemical wound environment, remain in place for the required treatment period but can be easily removed, and are acceptable to both patients and health care professionals.
- The only way for clinicians to fully trust wound dressing products is to develop clinically relevant laboratory test standards that result in comprehensive performance metrics for dressings.
- Clinically relevant testing standards would facilitate objective, standardized, and quantitative comparisons across wound dressing products and brands, leading to informed treatment decisions, and thereby to better patient outcomes.

## ABOUT THE AUTHORS

**Amit Gefen, PhD,** is a Professor with the Faculty of Engineering, Tel Aviv University, and the Berman Chair in Vascular Bioengineering.

**Paulo Alves, PhD,** is an Assistant Professor of Nursing and Tissue Viability at the Universidade Católica Portuguesa, Porto, Portugal.

**Dimitri Beeckman, PhD,** is a Professor of Nursing at Ghent University, Belgium and Örebro University, Sweden.

**Breda Cullen, PhD,** is a biochemist and independent consultant at RedC Consultancy.

**Jose Luis Lázaro-Martínez, PhD,** is a Professor at the Complutense University of Madrid, Spain.

**Hadar Lev-Tov, MD,** is an Assistant Professor at the University of Miami Department of Dermatology and Cutaneous Surgery, FL, USA.

**Bijan Najafi, PhD,** is a Professor and Director of Clinical Research at the Department of Surgery, Baylor College of Medicine, TX, USA.

**Nick Santamaria, PhD,** is a Professor of Nursing Research, Translational Research, Skin Integrity, and Wound Care at the University of Melbourne and the Royal Melbourne Hospital, Australia.

**Andrew Sharpe, MSc,** is a podiatrist working for the National Health Services of the United Kingdom.

**Terry Swanson, NP,** is a specialist in wound management working in a wound clinic in Warrnambool, Australia.

**Kevin Woo, PhD,** is a Professor at Queen's University, Schools of Nursing and Rehabilitation, Kingston, Canada.

## Abbreviations and Acronyms

HRV: heart rate variability
IWDTEP: International Wound Dressing Technology Expert Panel
MVTR: moisture-vapor transmission rate
PU: polyurethane
PVA: polyvinyl alcohol

## Notes

^†^
For example, in the TIME model—a widely used practical guide to wound management developed by a global team of wound care experts, which relates clinical observations and interventions to the underlying wound pathology in several aspects, including moisture imbalance.^15^

^‡^
Chronic wounds fail to heal because they do not progress from the proinflammatory state.

^§^
It is well established in the biomechanical engineering theory that stress concentrations in tissues occur due to sudden geometrical changes or irregularities in a contacting object, including sharp corners, which steeply increase the intensity of the local tissue stresses.^37^
